# Multiple Distinctive Demyelinating Lesions Caused by Eosinophilic Granulomatosis With Polyangiitis: Case Report and Literature Review

**DOI:** 10.3389/fneur.2019.00213

**Published:** 2019-03-11

**Authors:** Dingkang Xu, Hongen Xu, Fang Wang, Guoqing Wang, Qingjie Wei, Shixiong Lei, Qiang Gao, Qi Zhang, Fuyou Guo

**Affiliations:** ^1^Department of Neurosurgery, The First Affiliated Hospital of Zhengzhou University, Zhengzhou, China; ^2^Center for Precision Medicine, The First Affiliated Hospital of Zhengzhou University, Zhengzhou, China; ^3^School of Pharmaceutical Sciences, Zhengzhou University, Zhengzhou, China; ^4^Key Laboratory of Neurosurgical Diseases, The First Affiliated Hospital of Zhengzhou University, Zhengzhou, China

**Keywords:** EGPA, DNA sequencing, HLA, SNP, CNS involvement, neurosurgery, differential diagnosis

## Abstract

Eosinophilic granulomatosis with polyangiitis (EGPA) is an extremely rare rheumatic immune disease characterized by vasculitis of small- and medium-sized blood vessels. Central nervous system (CNS) involvement frequently consists of cerebrovascular disease; a manifestation with multiple demyelinating lesions has never been reported in detail. This report describes a 38-year-old man, who presented with progressive memory deterioration and underwent microsurgery; EGPA was subsequently confirmed. Unique clinical and radiological features as well as immunohistological outcomes and DNA sequencing revealed a potential disease-associated human leukocyte antigen (HLA) type, and single-nucleotide polymorphisms (SNPs) are described for this uncommon case. Although EGPA rarely involves the CNS, this differential diagnosis should be considered when patients present with a history of nasosinusitis, elevated eosinophil percentage, clinical pulmonitis, and neurological manifestations. Microsurgery is necessary for precise diagnosis and effective treatment.

## Introduction

Eosinophilic granulomatosis with polyangiitis (EGPA), an antineutrophil cytoplasmic antibody (ANCA)-associated vasculitis (AAV) previously known as Churg-Strauss syndrome, is characterized by an increase in peripheral and tissue eosinophilia. CNS involvement that includes multiple demyelinating lesions is uncommon with EGPA.

Here, we present the uncommon case of a male patient with memory loss who underwent microsurgery and a 10-month follow up and was subsequently diagnosed with EGPA. Whole-genome sequencing was performed. We report a brief overview of the clinical, radiological, and histological features associated with this rare disease. To the best of our knowledge, this case is the first instance of an EGPA patient with multiple lesions on imaging and unique demyelination.

## Case Presentation

### History and Examinations

A 38-year-old man presented with deterioration of memory, which had begun 1 month prior and was accompanied by impaired extension of the right upper limb. The patient had suffered from intermittent right frontal headache after catching a cold 10 days prior, during which hypomnesia was especially pronounced. His history included cholangiolithiasis, pancreatitis, and nasosinusitis. A metal biliary endoprosthesis had been placed endoscopically 8 months prior. The patient's family history was unremarkable. He was lucid but displayed poor comprehension, slow reaction time, decreased computational capabilities, and amnestic aphasia. Neurological examinations demonstrated no abnormalities except for impaired extension of the right upper extremity. Bilateral exophthalmos and cervical lymph node enlargement were found during the physical examination. The left upper eyelid was touching an active mass.

**Figure 1:**
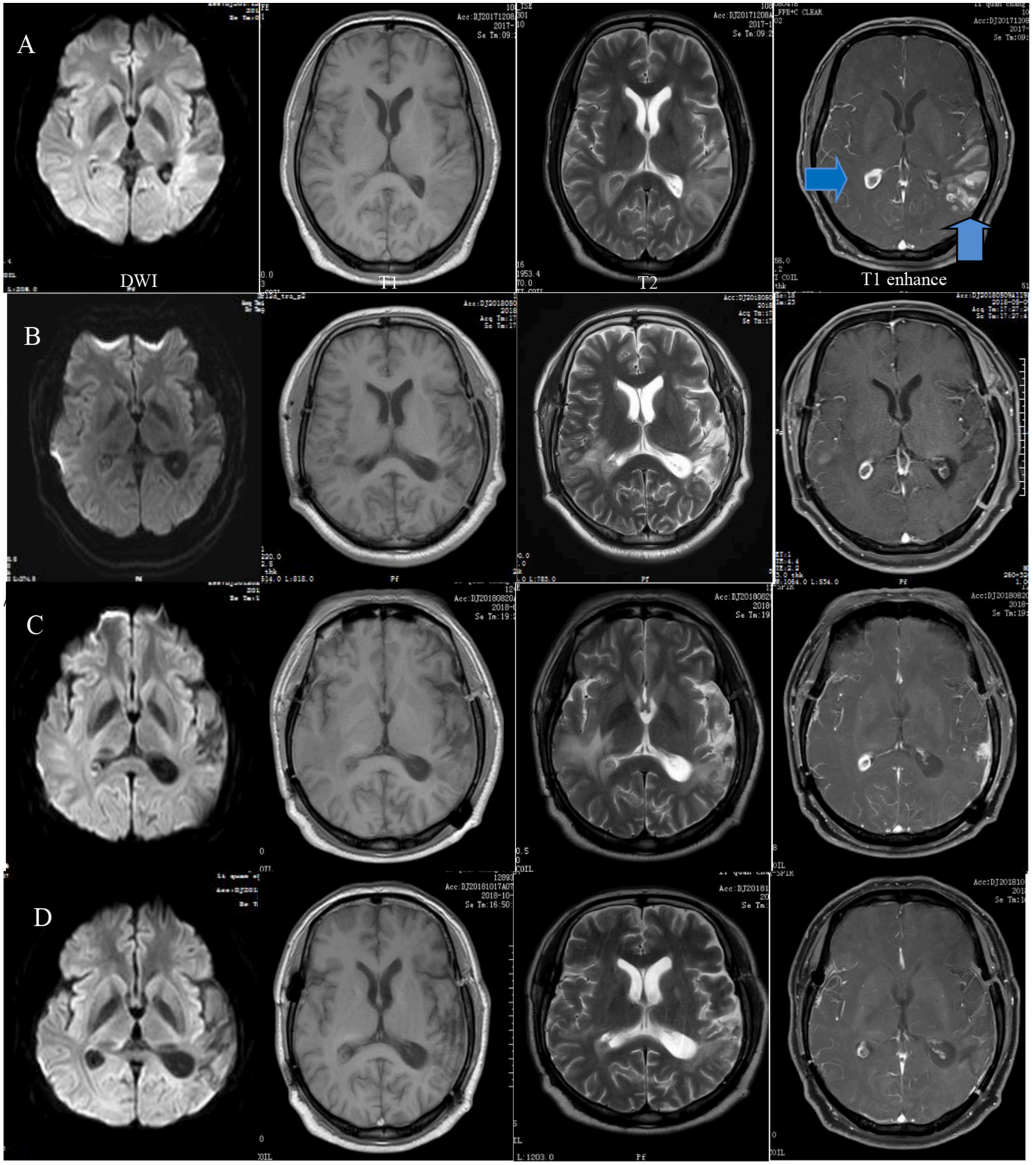
Preoperative MRI showing a wide range of long T1 and T2 signals in the left temporal, occipital, and insular lobes and diffusion-weighted imaging (DWI) demonstrating restricted diffusion in the same area as well as a right quasi-circular ring-enhancement after gadolinium administration **(A)**. A follow-up MRI 5 months after surgery demonstrated basically stable changes **(B)**. Eight months later, the extended range of edema became more apparent. Enhancement of the frontotemporal lobe was visible at the surgical site **(C)**. The range of edema and degree of enhancement were obviously reduced after 1 month of therapy **(D)**.

Laboratory tests for variables including tumor markers, relative levels of rheumatologically relevant antibodies, thyroid hormones, and routine blood parameters were all normal except for a rise in the erythrocyte sedimentation rate (ESR) to 46 mm/h and an elevated eosinophil percentage at 11.4%. Parasite infection was considered the primary diagnosis. Doppler ultrasound showed grade II enlargement of the cervical lymph nodes. Initial MRI and diffusion-weighted imaging (DWI) showed temporal and occipital lobe inflammation and colloid cysts in the right lateral ventricular trigone (Figure 1). Ocular MRI revealed increased volume of the bilateral tear glands, sinusitis in the entire group of paranasal sinuses and mastoiditis. Lumbar puncture indicated that the intracranial pressure was 170 mm H_2_O. The cerebrospinal fluid was sent out to be examined for cerebrospinal fluid-related viruses, rheumatic immune-related antibody, cerebrospinal fluid biochemistry, and autoimmune encephalitis antibodies. However, none of these tests were positive.

**Figure 2:**
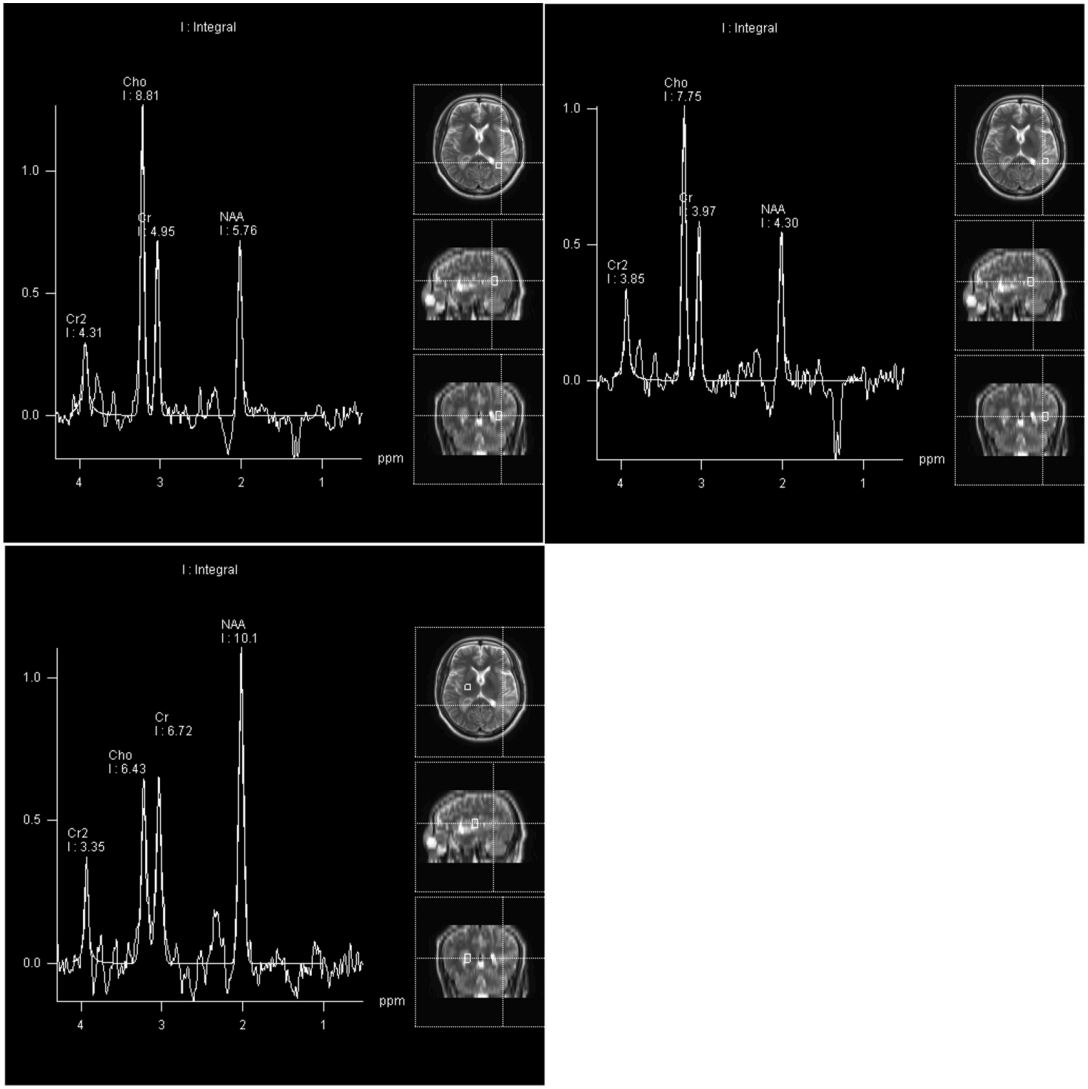
Compared with the right basal ganglia region as a normal control area, the NAA peak and NAA/Cr ratio in the pathological area of the left lateral ventricle trigone region were reduced, and the Cho peak and Cho/Cr ratio were increased.

Four days after admission, the patient began to cough. A CT scan showed bronchitis as well as inflammation of the left apex pulmonis and pleura. Two days later, extension of the right fingers suddenly became impaired. MR spectroscopy (MRS) demonstrated an obvious rise in choline (Cho) and a decrease in N-acetyl aspartate (NAA) in the left lateral ventricular trigone, indicating the possibility of a tumor lesion (Figure 2). We began to administer 120 mg methylprednisolone q.d. as an experimental therapy on day 9 of hospitalization, although the nature of the inflammation was unknown. On day 14 of hospitalization, the patient underwent microsurgery. Routine blood reexamination on the second day after the operation showed that the percentage of eosinophils had returned to normal. Except for a mild increase to 37.6°C 3 days after surgery, body temperature was normal. The patient quickly recovered, and his memory and right upper limb movement returned to normal 10 days after surgery.

The patient developed swelling of the right lower extremity 2 months after neurosurgery and was admitted to the hospital again in May 2018. Doppler ultrasound indicated thrombosis in the bilateral popliteal veins, right superficial femoral vein, anterior tibial vein, posterior tibial vein, and fibular vein. The percentage of eosinophils in peripheral blood had increased again to 14.8%. MRI reexamination showed stable enhancement of the right lateral ventricular trigone and normal postoperative changes in the left temporal-occipital lobe. The patient underwent percutaneous transluminal balloon dilatation and was discharged 5 days later.

Three months later, the patient came to the hospital for follow up. The disease seemed to have progressed this time. MRI showed a more pronounced degree of enhancement and a wider range of edema than before. A CT scan indicated double pneumonia and multiple enlarged lymph nodes in the mediastinum and under the axilla. The patient was then diagnosed with EGPA. He then received 1 g methylprednisolone p.o., q.d. for 3 days, and 60 mg prednisone q.d. was administered for 14 days, followed by 45 mg q.d. thereafter. Beginning 2 weeks after discharge from the hospital, 125 mg cyclophosphamide per day was taken orally. One month later, the symptoms had been alleviated, and MRI, CT, and Doppler ultrasound showed an excellent response to the treatment.

### Surgery

A subtotal resection of the right lateral ventricular trigone lesion was achieved via the subtemporal approach. The lesion was yellow-brown and unevenly textured and grew infiltratively in the shape of a cuff around the temporal-occipital artery. We performed piecemeal resection in the gliosis zone. Subsequently, the left temporo-occipital region was exposed. Multiple abnormal lesions around the Labbe vein showed infiltrative growth and abundant blood supply with soft material. Considering the presence of the lesion around the angular gyrus, subtotal resection was performed with careful manipulation.

### Histopathology and DNA Sequencing

**Figure 3:**
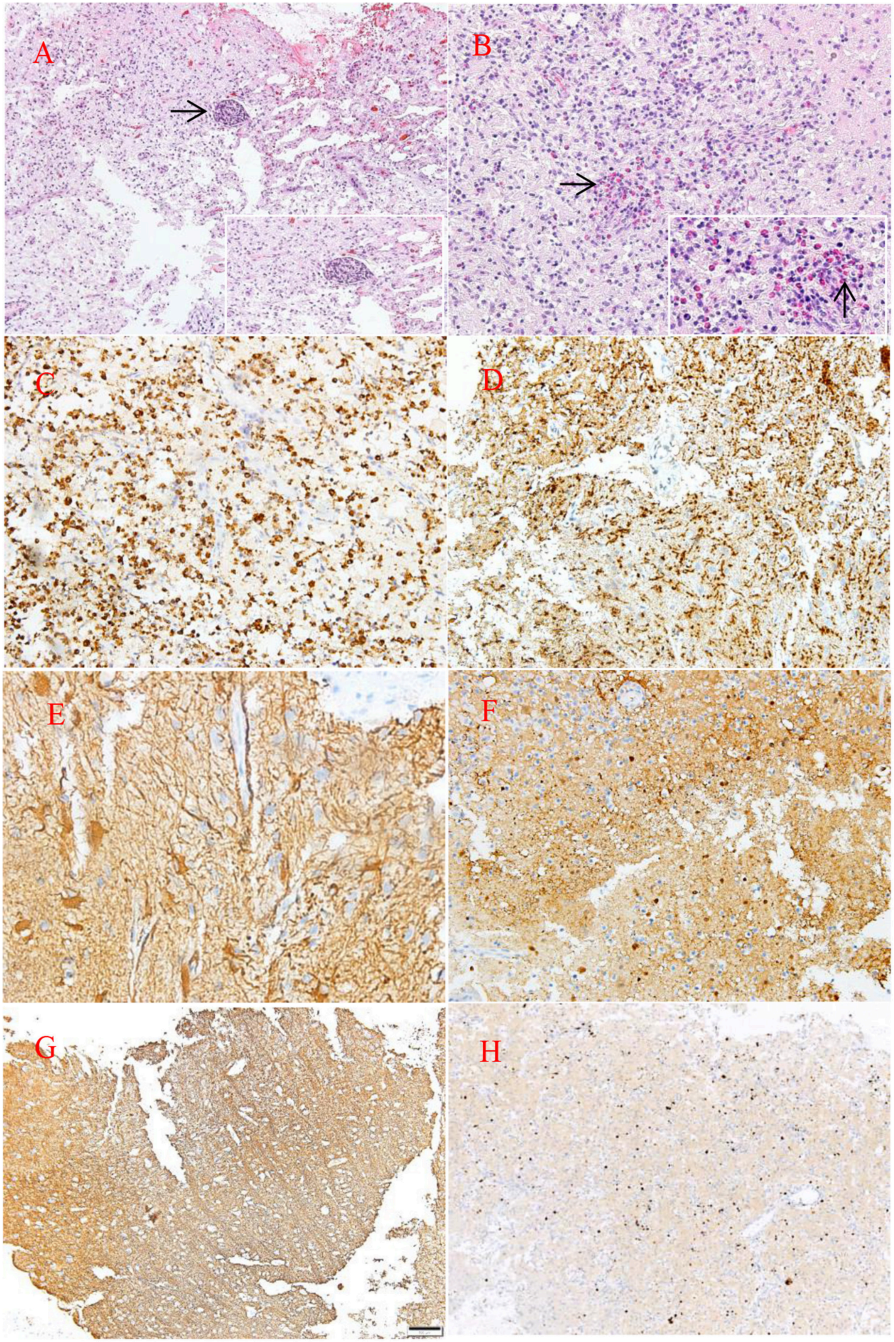
HE staining **(A,B)**. Pathological examination of the lesion revealed large quantities of histiocytes, lymphocytes, foam-like macrophages, perivascular lymphoid sheath (→), and infiltration of eosinophils (→). Neuraxon is clear in NF staining. Immunohistology showing the following: CD68(+), MBP(–), GFAP(+), S-100(+), NF(–), ki-67(10–20%) **(C,H)**. Original magnification × 10 **(A,F–H)** and × 20 **(B–E)**.

Hematoxylin and eosin staining of tumor specimens revealed inflammatory cell infiltration dominated by histiocyte and perivascular lymphoid sheath formation in the left temporo-occipital region. Microscopically, glial hyperplasia was observed in tissue from the right lateral ventricle trigone. Immunohistochemistry showed positivity for CD68, glial fibrillary acidic protein (GFAP), S-100, and synaptophysin (Syn) as well as focal positivity for Oligo-2. No reactivity was observed with antibodies against myelin basic protein (MBP), neurofilament (NF) or isocitrate dehydrogenase-1 (IDH-1). The MIB-1(ki-67) proliferation was approximately 10–20% (Figure 3).

**Figure 4:**
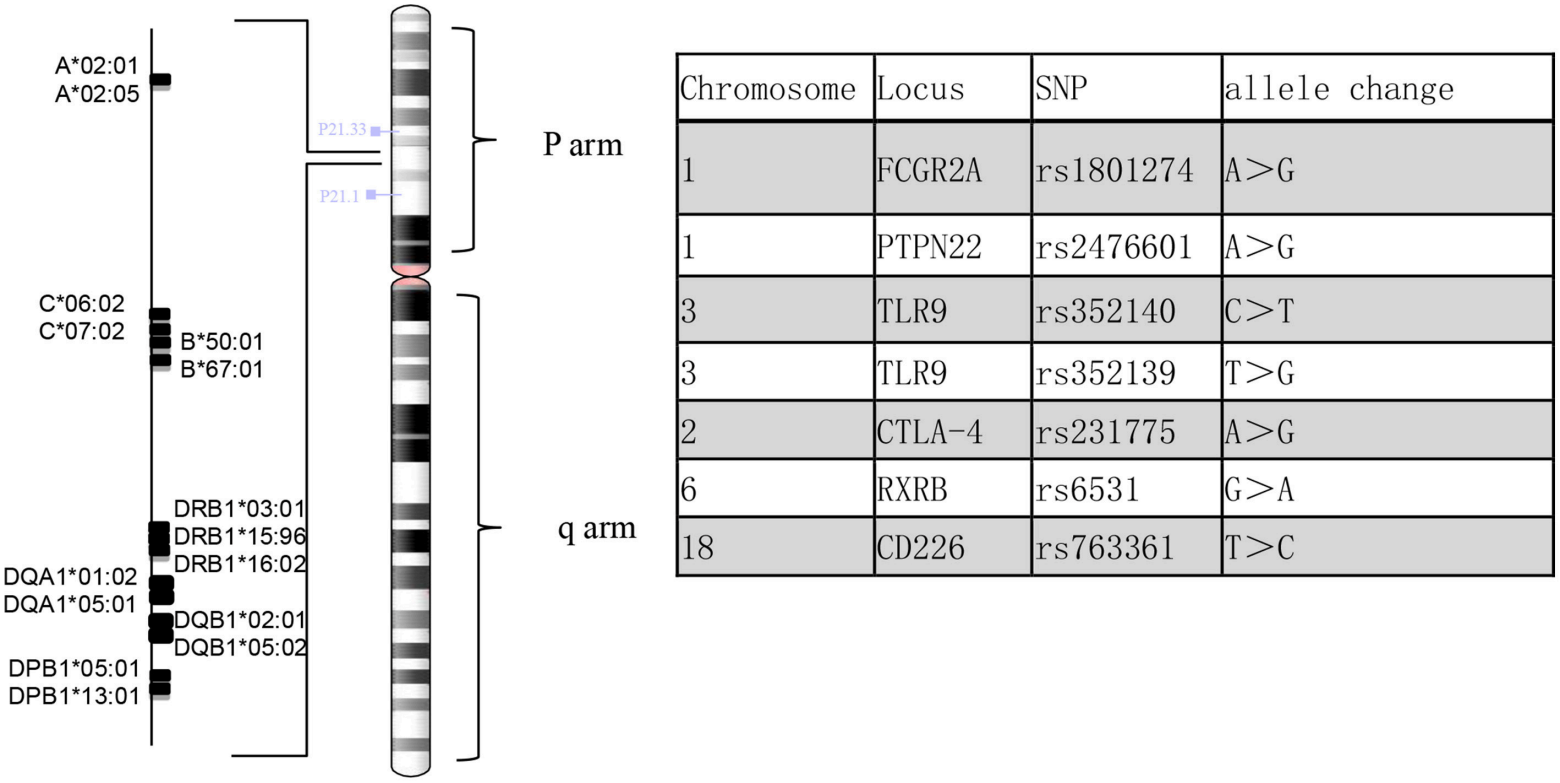
HLA alleles and altered SNPs in the present case.

Genomic DNA was extracted from the patient's peripheral blood using the QIAamp DNA Mini Kit (QIAGEN). Reads were mapped to the human reference genome assembly GRCh37 using BWA-MEM in the BWA package (version 0.7.17) with the default parameters. Human leukocyte antigen (HLA) genotyping was performed from the sequencing reads of the patient. We used three different genotyping algorithms, OptiType (1), BWAkit (https://github.com/iu-parfunc/bwa/tree/master/bwakit) and xHLA (2). Single nucleotide variants and indels were identified by the Freebayes tool (version 1.1.0) (https://github.com/ekg/freebayes). The SNPs in this patient were compared with those reported in the previous literature (3) (Figure 4).

## Discussion

EGPA belongs to the category of small- and medium-vessel AAVs, which are characterized by three overlapping disease processes: asthma and other allergy symptoms, tissue and blood eosinophilia, and necrotizing vasculitis (4, 5). Based on the presence or absence of ANCA, the association between phenotype and pathogenesis is unclear (4–7). ANCA-positive EGPA is especially likely to be correlated with peripheral neuropathy and glomerulonephritis, while the prognosis of ANCA-negative patients depends on cardiac involvement (4–6).

Nervous system involvement, especially peripheral mononeuropathy, is common, but CNS involvement is extremely rare. CNS involvement has been reported in 5.2 and 8.7% of EGPA patients retrospectively, whereas cerebral infarcts and subarachnoid hemorrhages are the main CNS manifestations reported in the literature (7–9). Raphaël André reported the largest study of EGPA describing CNS involvement with a low incidence of involvement before diagnosis (7). This report is the first detailed report to date of EGPA presenting as multiple lesions with multiple demyelinating impairments. In many patients, EGPA mainly affects the peripheral nerves, airways, gastrointestinal tract, heart, kidney, etc. However, the initial symptoms of this case were not typical, and the patient mainly presented CNS symptoms without other organ symptoms. Consequently, CNS neuropathy in EGPA should be considered in differential diagnoses. The laboratory test results and MRI findings suggested differential diagnoses of parasite infection cerebral sparganosis, tumefactive demyelinating lesions (TDLs), pulmonary *Aspergillus* infection, autoimmune encephalitis, mitochondrial encephalopathy, primary central system vasculitis, IgG4-related disease, etc. In GPA, cytoplasmic ANCA (c-ANCA) and proteinase 3-ANCA (PR3-ANCA) are relatively common, whereas perinuclear ANCA (p-ANCA) and myeloperoxidase-ANCA (MPO-ANCA) are more frequent in microscopic polyangiitis (MPA) and EGPA (5). Hypereosinophilic infiltration is an important feature of EGPA that distinguishes it from other AAVs. In our experience, Cho/NAA ratio is generally lower in TDLs than in glioma. Brain biopsy is essential for diagnosis of TDLs (10). A mass of myelin debris can be seen under microscopy in demyelinating diseases; we also observed irregular myelin morphology in a small field of view, which may have been caused by nerve ischemia due to eosinophilic infiltration of blood vessel walls (4). On account of the overlapping disease process, cellulose necrosis of vascular wall is not always found in pathological examinations. Aside from the negative effect of ANCA in this case, it is difficult to distinguish these conditions since the clinical symptoms and laboratory tests sometimes share overlapping characteristics. The AAVs are a group of syndromes that are subject to change over time as the disease develops in a given patient (5). Disease progression, MRS support of a tumor diagnosis and negative laboratory tests confused us and delayed the accurate diagnosis of the lesion. We did consider a diagnosis of EGPA at the first discharge when the patient's symptoms were relieved after surgery. Nevertheless, he did not receive standard glucocorticoid therapy, and the eosinophil evaluation was normal the second day after surgery, which is not in line with expectations. Moreover, there was no positive indication of ANCA-related antibody over his whole course of the disease.

The diagnosis of EGPA is based on the American College of Rheumatology 1990 criteria and the definition from the 2012 Chapel Hill Consensus Conference. As a subdivision of AAVs, the clinical diagnosis and associated ANCA expression do not meet the standard (3, 11). Whether AAVs represent subtypes of a single spectrum of totally different diseases is still an open question, but they may share a common genetic background in the reported literature (4, 11). Multiple studies have demonstrated the possibility of gene association with EGPA (3, 4, 6, 12), including gene variants in the major histocompatibility complex (MHC) region and inflammatory processes. At the moment, we also lack epidemiological data from Chinese EGPA patients, which is so rare that clinicians lack awareness; determining the genetic basis is urgent. In this case, DNA sequencing was performed in the MHC region (Figure 4). The SNPs on CTLA-4 (rs231775) and PTPN22 (rs2476601) were observed in patients with increased susceptibility to AAVs. Moreover, we observed MHC differences compared with the reference allele. HLA-B^*^67:01 is a genetic risk factor for Takayasu arteritis (13), while HLA-DRB1^*^16:02, HLA-DQB1^*^05:02 and HLA-B^*^67:01 are associated with relapsing polychondritis (RPC) (14), which may suggest a patient with a high susceptibility to another autoimmune disease (13). Interestingly, Chenan Zhang (15) reported that the DRB1^*^1501-DQA1^*^0102-DQB1^*^0602 haplotype may contribute to the risk of glioma in a non-additive manner. The type of role that DQA1^*^0102 plays in the process of disease pathogenesis is unclear. Future studies are needed to validate the significance of different allele associations.

To date, glucocorticoids and immunosuppressants remain the conventional treatment for EGPA (4, 6, 8, 9). Rituximab as an induction therapy to refractory EGPA proved effective in different cases (16, 17). Omalizumab, another Ig-E monoclonal antibody, improved asthma control in some patients with EGPA (17, 18). Recently, Wechsler reported that mepolizumab resulted in significantly more weeks in remission and reduced glucocorticoid use in a large cohort (19).

## Conclusion

In summary, EGPA manifesting as multiple unique demyelinating lesions is not a commonly observed presentation. The differential diagnosis of EGPA should be emphasized with demyelinating diseases and intracranial lesions such as gliomatosis and metastatic tumor. Pathological examination is necessary for diagnosis. In particular, patients can present with a history of nasosinusitis, an elevated eosinophil percentage, clinical pulmonitis, and neurological manifestation with multiple lesions. DNA sequencing is beneficial to providing evidence for the precise diagnosis of rare EGPA.

## Ethics Statement

The protocol was approved by the medical ethical committee of Zhengzhou University. This patient gave written informed consent in accordance with the Declaration of Helsinki. The authors affirm that written informed consent was obtained from the participant for the publication of this case report.

## Author Contributions

DX: conception, manuscript writing, and data analysis. HX: DNA sequencing analysis. FW and GW: data analysis. QW, SL, and QG: data acquisition. QZ and FG: conception and critical review. All authors proofread and approved the manuscript.

### Conflict of Interest Statement

The authors declare that the research was conducted in the absence of any commercial or financial relationships that could be construed as a potential conflict of interest.
